# *N*,*N*,*N*′,*N*′-Tetra­kis(2-hydroxy­ethyl)terephthalamide

**DOI:** 10.1107/S1600536808041573

**Published:** 2008-12-20

**Authors:** Zhi-Qiang Wang, Chen Xu, Ying-Fei Li, Fei-Fei Cen, Yu-Qing Zhang

**Affiliations:** a College of Chemistry and Chemical Engineering, Luoyang Normal University, Luoyang 471022, People’s Republic of China; b Chemical Engineering and Pharmaceutics School, Henan University of Science and Technology, Luoyang 471003, People’s Republic of China

## Abstract

The mol­ecule of the title compound, C_16_H_24_N_2_O_6_, which lies on a crystallographic inversion centre in the centre of the benzene ring, adopts an *anti* conformation in terms of the relative orientation of two amide carbonyl groups. One pair of the 2-hydroxy­ethyl groups is partially disordered with site occupancy factors of 0.811 (2) and 0.189 (2). The dihedral angle between the amide group and central benzene ring is 67.0 (2)°. Two O—H⋯O and one bifurcated O—H⋯(O,O) hydrogen bonds are present, resulting in a three-dimensional network.

## Related literature

For bond-length data, see: Allen *et al.* (1987 ▶). For general background, see: Katoono *et al.* (2006 ▶); Tosin *et al.* (2005 ▶); Yin *et al.* (2005 ▶). 
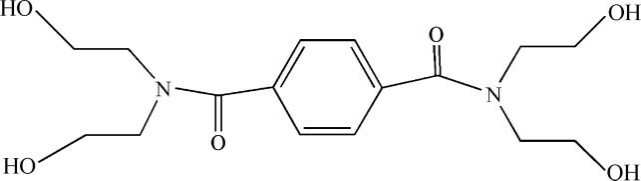


## Experimental

### 

#### Crystal data

C_16_H_24_N_2_O_6_
*M* *_r_* = 340.37
Orthorhombic, 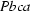

*a* = 10.3244 (12) Å
*b* = 12.5378 (14) Å
*c* = 12.8384 (15) Å
*V* = 1661.9 (3) Å^3^
*Z* = 4
Mo *K*α radiation
μ = 0.10 mm^−1^
*T* = 296 (2) K
0.29 × 0.24 × 0.23 mm

#### Data collection

Bruker SMART APEXII detector diffractometer
Absorption correction: multi-scan (*SADABS*; Sheldrick, 1996 ▶) *T*_min_ = 0.961, *T*_max_ = 0.976
11505 measured reflections
1550 independent reflections
1273 reflections with *I* > 2σ(*I*)
*R*_int_ = 0.022

#### Refinement

*R*[*F* ^2^ > 2σ(*F* ^2^)] = 0.042
*wR*(*F* ^2^) = 0.126
*S* = 1.06
1550 reflections
111 parameters
H-atom parameters constrained
Δρ_max_ = 0.35 e Å^−3^
Δρ_min_ = −0.21 e Å^−3^

### 

Data collection: *APEX2* (Bruker, 2004 ▶); cell refinement: *SAINT* (Bruker, 2004 ▶); data reduction: *SAINT*; program(s) used to solve structure: *SHELXS97* (Sheldrick, 2008 ▶); program(s) used to refine structure: *SHELXL97* (Sheldrick, 2008 ▶); molecular graphics: *SHELXTL* (Sheldrick, 2008 ▶); software used to prepare material for publication: *SHELXTL*.

## Supplementary Material

Crystal structure: contains datablocks global, I. DOI: 10.1107/S1600536808041573/rn2054sup1.cif

Structure factors: contains datablocks I. DOI: 10.1107/S1600536808041573/rn2054Isup2.hkl

Additional supplementary materials:  crystallographic information; 3D view; checkCIF report

## Figures and Tables

**Table 1:** Hydrogen-bond geometry (Å, °)

*D*—H⋯*A*	*D*—H	H⋯*A*	*D*⋯*A*	*D*—H⋯*A*
O2—H2*A*⋯O3^i^	0.82	1.91	2.723 (9)	169
O2—H2*A*⋯O3′^i^	0.82	1.90	2.675 (10)	157
O3′—H3′⋯O2^ii^	0.82	2.31	2.675 (3)	108
O3—H3*D*⋯O1^iii^	0.82	2.00	2.810 (2)	170

Symmetry codes: (i) 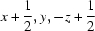; (ii) 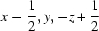; (iii) 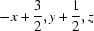.

**Fig. 1.**
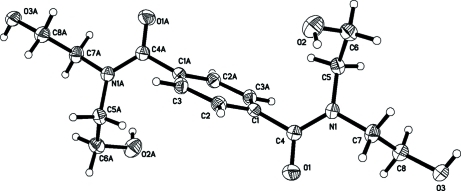
The molecular structure of the title compound with displacement ellipsoids at the 30% probability level (suffix A denotes the symmetry code: -x + 2, -y, -z + 1).

**Fig. 2.**
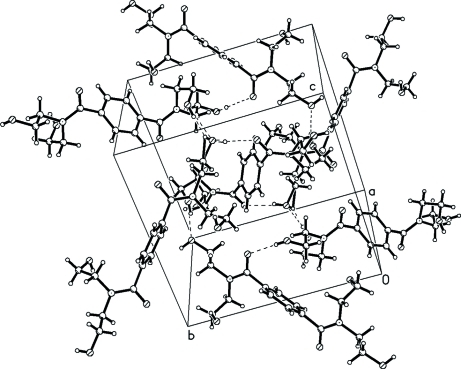
Partial view of the crystal packing showing the intermolecular O—H···O hydrogen bonds.

## supplementary crystallographic information

### Comment

Terephthalamide derivatives are important compounds in molecular recognition and supramolecular chemistry (Yin *et al.*, 2005; Tosin *et al.*,2005; Katoono *et al.*,2006). Although numerous tetrasubstituted terephthalamides have been investigated, only a few tetrakis(alkyl)terephthalamides are known. In order to further the study of such compounds, we report the crystal structure of the title compound.

A view of the molecular structure of the title compound is given in Fig.1. Molecules of the title compound lie across  crystallographic inversion centres and adopt the anti-conformation. The bond distances and angles are normal (Allen *et al.*, 1987). One set of the 2-hydroxyethyl groups is disordered with site occupancy factors of *ca* 0.811 (2) and 0.189 (2). The dihedral angle between the amide plane (C4,O1,N1) and phenyl planes (C1—C3,C1A—C3A) is 67.0 (2)°. The structural study shows the presence of four different intermolecular O—H···O hydrogen bonds (Table 1), resulting in a three-dimensional supramolecular architecture (Fig. 2).

### Experimental

To a solution of diethanolamine (2 mmol) in dry chloroform (5 ml), at 273 K, was added dropwise a solution of terephthalyl chloride (2 mmol) in dry chloroform (25 ml). Then, the mixture stirred at room temperature for 24hr, removal of solvent resulted in a yellow powder that was recrystallized from methanol-DMF solution at room temperature to give the desired product as colourless crystals suitable for single-crystal X-ray diffraction.

### Refinement

H atoms attached to C atoms of the title compound were placed in geometrically idealized positions and treated as riding with C—H distances constrained to 0.93–0.97 Å, with *U*ĩso~(H) = 1.2 or 1.5 times *U*~eq~(C). H atoms bonded to O atoms were located in a difference map and refined independently with isotropic displacement parameters.

### Crystal data

**Table:**

C_16_H_24_N_2_O_6_	*D*_x_ = 1.360 Mg m^−^^3^
*M**_r_* = 340.37	Mo *K*α radiation, λ = 0.71073 Å
Orthorhombic, *P**b**c**a*	Cell parameters from 3593 reflections
*a* = 10.3244 (12) Å	θ = 3.0–23.6°
*b* = 12.5378 (14) Å	µ = 0.10 mm^−^^1^
*c* = 12.8384 (15) Å	*T* = 296 K
*V* = 1661.9 (3) Å^3^	Block, colourless
*Z* = 4	0.29 × 0.24 × 0.23 mm
*F*(000) = 728	

### Data collection

**Table:**

Bruker SMART APEXII detector diffractometer	1550 independent reflections
Radiation source: fine-focus sealed tube	1273 reflections with *I* > 2σ(*I*)
graphite	*R*_int_ = 0.022
phi and ω scans	θ_max_ = 25.5°, θ_min_ = 3.0°
Absorption correction: multi-scan (*SADABS*; Sheldrick, 1996)	*h* = −12→12
*T*_min_ = 0.961, *T*_max_ = 0.976	*k* = −15→15
11505 measured reflections	*l* = −15→15

### Refinement

**Table:**

Refinement on *F*^2^	Primary atom site location: structure-invariant direct methods
Least-squares matrix: full	Secondary atom site location: difference Fourier map
*R*[*F*^2^ > 2σ(*F*^2^)] = 0.042	Hydrogen site location: inferred from neighbouring sites
*wR*(*F*^2^) = 0.126	H-atom parameters constrained
*S* = 1.06	*w* = 1/[σ^2^(*F*_o_^2^) + (0.061*P*)^2^ + 0.6392*P*] where *P* = (*F*_o_^2^ + 2*F*_c_^2^)/3
1550 reflections	(Δ/σ)_max_ < 0.001
111 parameters	Δρ_max_ = 0.35 e Å^−^^3^
0 restraints	Δρ_min_ = −0.21 e Å^−^^3^

### Special details

**Table:**

Geometry. All e.s.d.'s (except the e.s.d. in the dihedral angle between two l.s. planes)are estimated using the full covariance matrix. The cell e.s.d.'s are takeninto account individually in the estimation of e.s.d.'s in distances, anglesand torsion angles; correlations between e.s.d.'s in cell parameters are onlyused when they are defined by crystal symmetry. An approximate (isotropic)treatment of cell e.s.d.'s is used for estimating e.s.d.'s involving l.s. planes.
Refinement. Refinement of *F*^2^ against ALL reflections. The weighted *R*-factor *wR* andgoodness of fit *S* are based on *F*^2^, conventional *R*-factors *R* are basedon *F*, with *F* set to zero for negative *F*^2^. The threshold expression of*F*^2^ > σ(*F*^2^) is used only for calculating *R*-factors(gt) *etc*. and isnot relevant to the choice of reflections for refinement. *R*-factors basedon *F*^2^ are statistically about twice as large as those based on *F*, and *R*-factors based on ALL data will be even larger.

### Fractional atomic coordinates and isotropic or equivalent isotropic displacement parameters (Å^2^)

**Table:**

	*x*	*y*	*z*	*U*_iso_*/*U*_eq_	Occ. (<1)
C7	0.78238 (19)	0.31459 (17)	0.30476 (16)	0.0460 (6)	0.811 (2)
H7A	0.8162	0.3857	0.3168	0.055*	0.811 (2)
H7B	0.8130	0.2906	0.2373	0.055*	0.811 (2)
C8	0.63782 (13)	0.31829 (14)	0.30437 (14)	0.0498 (6)	0.811 (2)
H8A	0.6033	0.2481	0.2887	0.060*	0.811 (2)
H8B	0.6064	0.3396	0.3725	0.060*	0.811 (2)
O3	0.59529 (19)	0.39348 (12)	0.22709 (12)	0.0569 (6)	0.811 (2)
H3D	0.6203	0.4534	0.2429	0.085*	0.811 (2)
C7'	0.70527 (19)	0.28471 (18)	0.34485 (17)	0.0460 (6)	0.189 (2)
H7'1	0.6561	0.3216	0.3982	0.055*	0.189 (2)
H7'2	0.6523	0.2288	0.3147	0.055*	0.189 (2)
C8'	0.75511 (17)	0.36199 (17)	0.26151 (16)	0.0498 (6)	0.189 (2)
H8'1	0.8070	0.4170	0.2944	0.060*	0.189 (2)
H8'2	0.8102	0.3236	0.2131	0.060*	0.189 (2)
O3'	0.6539 (2)	0.40926 (16)	0.20721 (15)	0.0569 (6)	0.189 (2)
H3'	0.5952	0.3659	0.1996	0.085*	0.189 (2)
C1	0.93464 (15)	0.07120 (12)	0.43518 (12)	0.0362 (4)	
C2	1.06224 (16)	0.04283 (12)	0.41398 (13)	0.0392 (4)	
H2	1.1041	0.0714	0.3562	0.047*	
C3	1.12701 (16)	−0.02751 (13)	0.47837 (14)	0.0405 (4)	
H3	1.2124	−0.0458	0.4638	0.049*	
C4	0.86624 (17)	0.14222 (13)	0.35884 (14)	0.0436 (4)	
C5	0.84524 (17)	0.28318 (13)	0.49384 (14)	0.0440 (4)	
H5A	0.8715	0.2257	0.5398	0.053*	
H5B	0.7615	0.3087	0.5175	0.053*	
C6	0.94175 (18)	0.37226 (15)	0.50233 (16)	0.0492 (5)	
H6A	0.9164	0.4296	0.4559	0.059*	
H6B	0.9410	0.3999	0.5729	0.059*	
N1	0.83155 (14)	0.24090 (12)	0.38745 (12)	0.0496 (4)	
O1	0.84678 (17)	0.10814 (11)	0.26939 (11)	0.0669 (5)	
O2	1.06824 (13)	0.33898 (13)	0.47707 (12)	0.0645 (5)	
H2A	1.0808	0.3474	0.4145	0.097*	

### Atomic displacement parameters (Å^2^)

**Table:**

	*U*^11^	*U*^22^	*U*^33^	*U*^12^	*U*^13^	*U*^23^
C7	0.0484 (13)	0.0439 (12)	0.0455 (12)	0.0069 (9)	−0.0011 (9)	0.0099 (9)
C8	0.0524 (13)	0.0459 (12)	0.0510 (13)	0.0052 (10)	−0.0096 (10)	0.0017 (10)
O3	0.0677 (14)	0.0500 (9)	0.0530 (11)	0.0135 (9)	−0.0240 (9)	−0.0022 (7)
C7'	0.0484 (13)	0.0439 (12)	0.0455 (12)	0.0069 (9)	−0.0011 (9)	0.0099 (9)
C8'	0.0524 (13)	0.0459 (12)	0.0510 (13)	0.0052 (10)	−0.0096 (10)	0.0017 (10)
O3'	0.0677 (14)	0.0500 (9)	0.0530 (11)	0.0135 (9)	−0.0240 (9)	−0.0022 (7)
C1	0.0407 (9)	0.0283 (8)	0.0397 (9)	0.0003 (6)	−0.0054 (7)	−0.0037 (7)
C2	0.0428 (9)	0.0343 (8)	0.0405 (9)	−0.0014 (7)	0.0026 (7)	0.0014 (7)
C3	0.0340 (8)	0.0362 (8)	0.0514 (10)	0.0022 (7)	0.0006 (7)	−0.0013 (7)
C4	0.0482 (10)	0.0382 (9)	0.0445 (9)	0.0042 (7)	−0.0087 (8)	−0.0018 (7)
C5	0.0449 (9)	0.0377 (9)	0.0494 (10)	0.0065 (7)	0.0000 (7)	−0.0032 (8)
C6	0.0548 (11)	0.0423 (10)	0.0505 (10)	−0.0013 (8)	0.0010 (9)	−0.0028 (8)
N1	0.0605 (10)	0.0382 (8)	0.0500 (9)	0.0135 (7)	−0.0178 (7)	−0.0031 (7)
O1	0.1004 (12)	0.0534 (8)	0.0468 (8)	0.0175 (7)	−0.0234 (8)	−0.0089 (6)
O2	0.0476 (8)	0.0816 (11)	0.0641 (9)	−0.0001 (7)	0.0030 (7)	0.0111 (8)

### Geometric parameters (Å, °)

**Table:**

C7—C8	1.4933 (14)	C1—C3^i^	1.392 (2)
C7—N1	1.496 (2)	C1—C4	1.501 (2)
C7—H7A	0.9700	C2—C3	1.382 (2)
C7—H7B	0.9700	C2—H2	0.9300
C8—O3	1.4372 (14)	C3—C1^i^	1.392 (2)
C8—H8A	0.9700	C3—H3	0.9300
C8—H8B	0.9700	C4—O1	1.242 (2)
O3—H3D	0.8200	C4—N1	1.339 (2)
C7'—N1	1.517 (2)	C5—N1	1.472 (2)
C7'—C8'	1.5324 (15)	C5—C6	1.501 (3)
C7'—H7'1	0.9700	C5—H5A	0.9700
C7'—H7'2	0.9700	C5—H5B	0.9700
C8'—O3'	1.3890 (13)	C6—O2	1.409 (2)
C8'—H8'1	0.9700	C6—H6A	0.9700
C8'—H8'2	0.9700	C6—H6B	0.9700
O3'—H3'	0.8200	O2—H2A	0.8200
C1—C2	1.391 (2)		
			
C8—C7—N1	111.14 (14)	C3—C2—C1	120.30 (16)
C8—C7—H7A	109.4	C3—C2—H2	119.8
N1—C7—H7A	109.4	C1—C2—H2	119.8
C8—C7—H7B	109.4	C2—C3—C1^i^	120.47 (15)
N1—C7—H7B	109.4	C2—C3—H3	119.8
H7A—C7—H7B	108.0	C1^i^—C3—H3	119.8
O3—C8—C7	109.1	O1—C4—N1	121.89 (16)
O3—C8—H8A	109.9	O1—C4—C1	118.42 (15)
C7—C8—H8A	109.9	N1—C4—C1	119.66 (15)
O3—C8—H8B	109.9	N1—C5—C6	113.53 (15)
C7—C8—H8B	109.9	N1—C5—H5A	108.9
H8A—C8—H8B	108.3	C6—C5—H5A	108.9
N1—C7'—C8'	101.08 (14)	N1—C5—H5B	108.9
N1—C7'—H7'1	111.6	C6—C5—H5B	108.9
C8'—C7'—H7'1	111.6	H5A—C5—H5B	107.7
N1—C7'—H7'2	111.6	O2—C6—C5	112.23 (15)
C8'—C7'—H7'2	111.6	O2—C6—H6A	109.2
H7'1—C7'—H7'2	109.4	C5—C6—H6A	109.2
O3'—C8'—C7'	111.6	O2—C6—H6B	109.2
O3'—C8'—H8'1	109.3	C5—C6—H6B	109.2
C7'—C8'—H8'1	109.3	H6A—C6—H6B	107.9
O3'—C8'—H8'2	109.3	C4—N1—C5	124.16 (14)
C7'—C8'—H8'2	109.3	C4—N1—C7	117.81 (15)
H8'1—C8'—H8'2	108.0	C5—N1—C7	117.94 (14)
C8'—O3'—H3'	109.5	C4—N1—C7'	117.74 (16)
C2—C1—C3^i^	119.23 (15)	C5—N1—C7'	106.66 (15)
C2—C1—C4	118.00 (15)	C7—N1—C7'	39.54 (8)
C3^i^—C1—C4	122.62 (15)	C6—O2—H2A	109.5
			
N1—C7—C8—O3	177.43 (15)	C1—C4—N1—C7	170.75 (15)
N1—C7'—C8'—O3'	177.19 (14)	O1—C4—N1—C7'	37.8 (3)
C3^i^—C1—C2—C3	−0.3 (3)	C1—C4—N1—C7'	−144.29 (16)
C4—C1—C2—C3	−176.06 (15)	C6—C5—N1—C4	113.8 (2)
C1—C2—C3—C1^i^	0.3 (3)	C6—C5—N1—C7	−62.8 (2)
C2—C1—C4—O1	64.1 (2)	C6—C5—N1—C7'	−104.03 (17)
C3^i^—C1—C4—O1	−111.5 (2)	C8—C7—N1—C4	98.8 (2)
C2—C1—C4—N1	−113.88 (19)	C8—C7—N1—C5	−84.3 (2)
C3^i^—C1—C4—N1	70.5 (2)	C8—C7—N1—C7'	−1.98 (10)
N1—C5—C6—O2	−63.2 (2)	C8'—C7'—N1—C4	−104.2 (2)
O1—C4—N1—C5	176.24 (18)	C8'—C7'—N1—C5	110.73 (19)
C1—C4—N1—C5	−5.8 (3)	C8'—C7'—N1—C7	−3.21 (8)
O1—C4—N1—C7	−7.2 (3)		

Symmetry codes: (i) −*x*+2, −*y*, −*z*+1.

### Hydrogen-bond geometry (Å, °)

**Table:**

*D*—H···*A*	*D*—H	H···*A*	*D*···*A*	*D*—H···*A*
O2—H2A···O3^ii^	0.82	1.91	2.723 (9)	169
O2—H2A···O3'^ii^	0.82	1.90	2.675 (10)	157
O3'—H3'···O2^iii^	0.82	2.31	2.675 (3)	108
O3—H3D···O1^iv^	0.82	2.00	2.810 (2)	170

Symmetry codes: (ii) *x*+1/2, *y*, −*z*+1/2; (iii) *x*−1/2, *y*, −*z*+1/2; (iv) −*x*+3/2, *y*+1/2, *z*.
